# SpiderPhy dataset: A multimodal dataset of Physiological, Psychometric and Behavioral Responses to fear stimuli

**DOI:** 10.1038/s41597-025-04908-x

**Published:** 2025-04-10

**Authors:** Cindy S. Lor, David Steyrl, Alexander Karner, Sebastian J. Götzendorfer, Anne Klimesch, Stephanie J. Eder, Fabian M. Renz, Johannes Rother, Frank Scharnowski, Filip Melinscak

**Affiliations:** 1 Department of Cognition, Emotion, and Methods in Psychology, University of Vienna, Vienna, Austria; 2 Zurich Center for Neuroeconomics, University of Zurich, Zurich, Switzerland

**Keywords:** Human behaviour, Physiology, Anxiety

## Abstract

The SpiderPhy dataset is a dataset of physiological, psychometric, and behavioral data from subclinical spider fearful individuals (N = 54) who underwent exposure to spider stimuli. The physiological data comprises electrocardiograms, electrodermal activity, respiration patterns, and oculometry data including gaze position and pupil size. After each spider-exposure trial, the stimulus was rated on a 0–100 scale on how much fear the stimulus evoked. Demographical data and behavioral data, including pre-post assessment of phobic symptoms, state of exhaustion, or experience of disgust, are also included. This dataset is of particular interest for researchers interested in investigating relationships between multiple physiological responses, current state of fear and/or phobic symptom severity.

## Background & Summary

Specific phobia is a prevalent and distressing anxiety disorder experienced by a substantial portion of the population. Exposure therapy, recognized as a primary treatment approach for specific phobias, involves the systematic and controlled confrontation of feared stimuli, offering individuals an opportunity to mitigate their phobic responses within a therapeutic context^1,2^. While the effectiveness of exposure therapy in clinical settings has been well-established, dropout rates are considerably high^1,3,4^, not all patients benefit from it^1,3^ and relapses after initial treatment success are frequent^5^.

Excessive distress during exposure has been hypothesized as a contributing factor to dropout rates and treatment ineffectiveness. Part of the therapist’s responsibility during therapy is to ensure that the exposure is appropriately challenging without causing excessive distress, while also ensuring that the patient remains cognitively engaged to reactivate their fear memory effectively. Traditionally, therapists rely on interpretation of bodily responses and questions to gauge distress levels, but there is a growing trend towards utilizing physiological signals to assist therapists in adapting the exposure stimulus to an appropriate fear-inducing level^6–9^. This is particularly relevant in novel therapy strategies, including those based on Virtual Reality Exposure Therapy^10,11^ or computerized interventions^12^, where comprehensive insights into the physiological responses occurring during exposure are a valuable source of information to infer the distress state of the patient so that the treatment is neither overwhelming nor underwhelming.

This dataset, collected from a cohort of N = 54 individuals with spider fear, consists of physiological responses and psychological ratings of 174 spider-related stimuli and 16 neutral stimuli. The physiological data include electrocardiogram (ECG) recordings, electrodermal activity (EDA) (or skin conductance responses (SCR)) recorded via electrodes, respiration rate and amplitude as monitored by a respiration belt and eye-tracking information such as gaze position and pupil size. The stimuli used to evoke fear, which have been all luminance-matched, are also available in this dataset.

Subjective psychological ratings of each picture pictures were also acquired, with participants providing fear and disgust ratings on a 0–100 scale following each exposure to spider stimuli.

Additional self-reports of fear, agitation, disgust, boredom, and exhaustion on a 0–10 scale were collected during breaks throughout the experiment.

The dataset also includes demographic data (age and gender) and a series of pre- and post-exposure assessments, i.e., the Fear of Spiders Questionnaire (FSQ)^13^, the Spider Phobia Questionnaire (SPQ)^14^, and the Spinnenangst Screening (SAS)^15^, which all provide standardized measures of phobic symptom severity. A fourth questionnaire, the Trait-section of the State-Trait Anxiety Inventory (STAI-T)^16^, was collected to provide additional aspects of participants’ anxiety profiles.

The SpiderPhy dataset offers significant potential for research in areas, such as investigating multimodal fear processing, developing machine learning models to predict fear responses, and advancing personalized exposure therapy by linking physiological markers to subjective fear ratings. It can also be used to study the temporal dynamics of fear responses and enables exploration of the relationships between trait anxiety, phobia severity, and physiological responses, providing valuable insights for both basic and applied research.

## Methods

### Subjects

Subjects were recruited via advertisement on Facebook and on the webpage of the University of Vienna. The inclusion criterion was having moderate to severe fear of spiders on the FSQ (FSQ >  = 24; the cut-off value corresponds to the midpoint score of the moderate fear group as defined by Cochrane *et al*.^17^). Exclusion criteria included non-corrected visual impairment and visual impairment corrected with glasses instead of contact lenses, past or present neurological or psychiatric conditions (other than spider phobia), current pregnancy, and current or past excessive alcohol or drug consumption. Compensation consisted of either seven course credits or 20 euros. A final sample of N = 54 spider-fearful individuals aged 18 to 30 years completed the experiment at some point between November 2019 and February 2020. Descriptive statistics and the results of the pre-questionnaire are shown in Table 1. All the participants provided written informed consent. The study was approved by the Ethics Committee of the University of Vienna (reference number: 00479). The whole experiment was performed in German.

**Table 1:** Descriptive statistics.

Variable	Mean ± Std
FSQ pre-exposure	54.4 + 19.4
FSQ post-exposure	52.0 + 24.0
SPQ	14.7 ± 5.9
SAS pre-exposure	17.0 ± 4.0
SAS post-exposure	14.8 ± 4.9
STAI-T	41.0 ± 9.9
Gender	8 M; 46 F
Age	21.6 ± 3.0

### Setup and procedure

The experiment consisted of the exposure to a set of 174 spider-related stimuli and 16 neutral stimuli (inanimate objects like a chair, a bike…) presented on a computer screen using PsychoPy 3.2.4. The exposure was divided into 4 blocks of about 56 trials each. Each trial consisted of a fixation cross (3 to 5 seconds), followed by a picture from the dataset (5 sec) and a rating phase, where participants indicated their level of fear on a continuous scale (0–100) by moving a cursor with a mouse. The left and right extremes of the rating scale were defined as ‘no fear’ and ‘a lot of fear’, respectively. Each block was preceded by a 90-second relaxation phase and followed by a short break during which the participants filled out questionnaires. An additional 17 of the images were presented a second time to each participant to allow for reliability testing (208 pictures shown in total). 16 catch-trials were inserted throughout the experiment, for which the participants were instructed to move the mouse to either the very left or right of the scale instead of providing ratings.

**Fig. 1:**
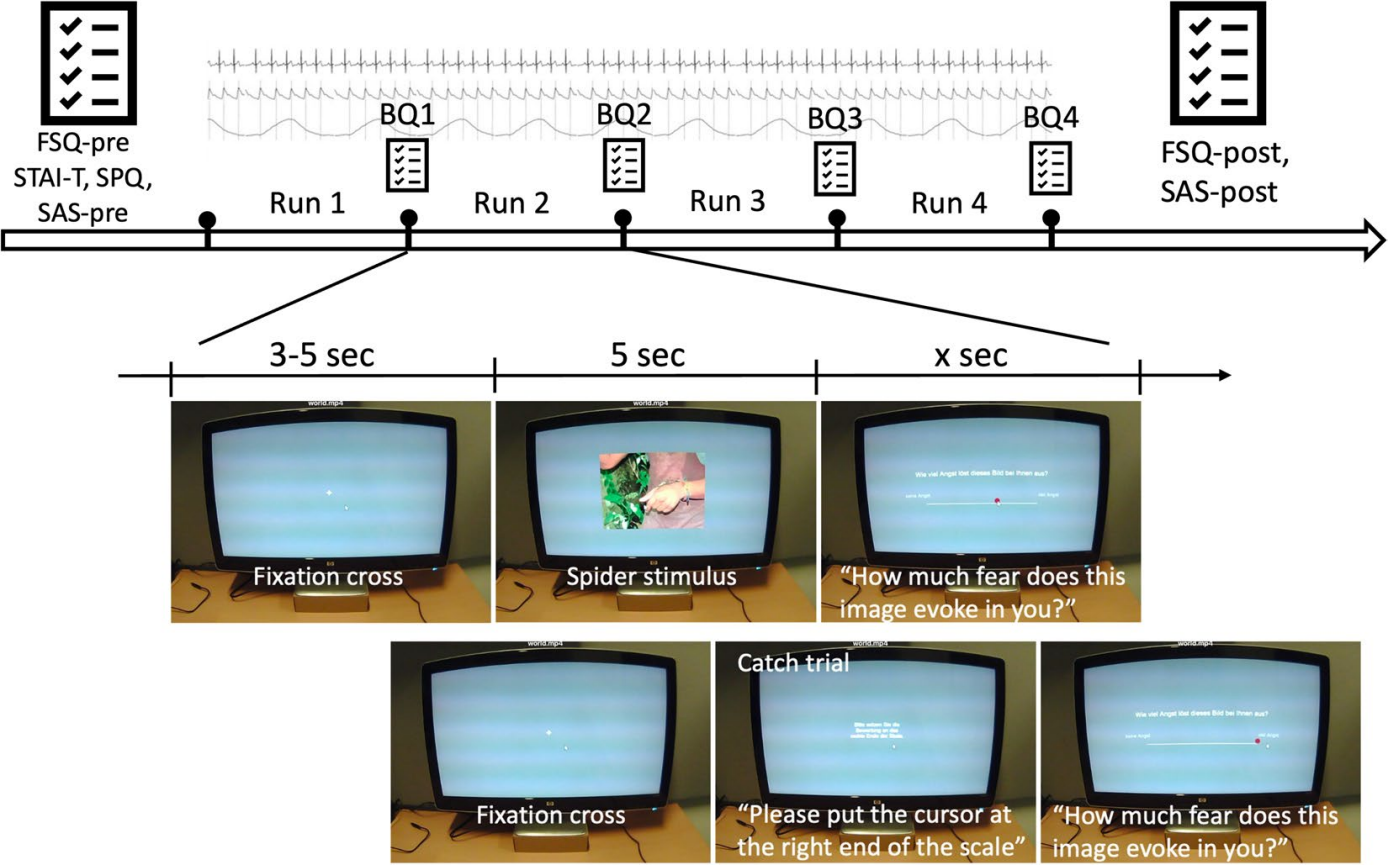
Experimental procedure. Participants were exposed to pictures of spiders and asked to rate how much fear the picture has evoked. Non-spider pictures and catch trials were also inserted in the experiment. The experiment was divided into 4 runs of 56 trials each and after each run, the participants filled brief questionnaires on their current psychological state. At the beginning and at the end of the session, they filled several questionnaires to evaluate the severity of their spider phobia (Fear of Spider Questionnaire (FSQ) and Spinnenangst-Screening (SAS)). The State-Trait Anxiety Inventory - Trait (STAI-T) and the Spider Phobia Questionnaire (SPQ) were collected at the beginning of the experiment only. The experiment was conducted in German.

An experimental session consisted of a briefing, the setup and quality checks for all physiological measurements, a practice block with five neutral pictures, 4 blocks of spider exposure interleaved with three breaks, post-exposure questionnaires and a short debriefing. The whole procedure took approximately 1.5 hours per participant. See Fig. 1 for an overview.

### Stimuli

The stimuli set are a part of larger research project “SpiDa” on spider fear stimuli^18^ and consists of pictures taken from the Geneva Affective Picture Database (GAPED)^19^ (https://www.unige.ch/cisa/research/materials-and-online-research/research-material/), from free stock photography websites (search terms: “spider”, “spiders”, “cobweb”, “cobwebs”, “arachnid”, “arachnids”, “cartoon spider”, “spider web”), as well as of self-taken images. For this experiment, mean luminance of images was normalized in the hue, saturation, lightness (HSV) color space (Mhsv = 0.5615, SDhsv = 0.2466) and the CIELAB color space (Mlab = 728.687, SDlab = 174.884). Luminance matching was performed with the Spectrum, Histogram, and Intensity Normalization and Equalization (SHINE) color toolbox^20^ which was adapted from the SHINE toolbox^21^. The images were also adjusted for size (600 × 800 pixels). The lightning conditions of the room were kept constant across all data acquisitions. The luminance-corrected ‘spiderPhy’ stimuli are publicly available in a separate repository: https://osf.io/vmuza^22^.

### Physiological measurements

Four physiological measurements were simultaneously collected during the exposure and picture-rating task.

The lighting conditions of the room were kept constant across all data acquisitions by closing the blinds and keeping the regular LED office light at approx. 400 lux and a color temperature of 4500 K (neutral white light). The room temperature was kept at approximately 21 degrees Celsius.

1. *Pupil dilation*: A binocular, mobile eye tracker (‘Pupil Core’, Pupil Labs, Berlin, Germany; Kassner *et al*., 2004) was employed to record the pupil sizes of both eyes following the user guide of the manufacturer (https://docs.pupil-labs.com/core/software/pupil-capture/). The participant was placed about 80 cm away from the screen. We asked the participants to avoid moving their head.
2. *Respiration*: A respiration belt (BrainProducts GmbH, Gilching, Germany) was attached in the lower chest/upper abdominal area to measure breathing patterns.
3. *Galvanic skin conductance*: Electrodermal activity electrodes (Ag/AgCl, BrainProducts GmbH, Gilching, Germany) were applied to the intermediate phalanges of 2nd and 3rd digits of the inner non-dominant hand and secured with finger straps. Prior to attaching the electrodes, the skin was cleaned with alcohol and an isotonic GSR electrolyte gel was applied to ensure connection between skin and electrodes.
4. *Electrocardiography (ECG)*: ECG signals were recorded using Biopac EL500Series disposable electrodes (Biopac, Los Angeles, USA). Skin preparation and electrode placement followed the procedure described in the Biopac ECG guidelines (https://www.biopac.com/wp-content/uploads/ECG-Guide.pdf). The skin at the electrode sites was cleaned with Biopac skin prep gel, and any nearby jewelry was removed. Electrode gel (GEL100, Biopac) was placed on the electrode before attaching it to the skin and surgical tape was used to hold the electrode leads in place. We recorded a Lead II configuration which measures the voltage between the right arm and left leg. Electrodes were placed on the inner left ankle (plus pole) and the inner right wrist (minus pole), with a ground electrode on the right ankle.

Additionally, a pulse oximeter (Nellcor, Minneapolis, USA) was placed on the 4th digit of the non-dominant hand. However, since this signal provided data akin to the ECG, we did not utilize its readings in our analyses.

ECG, Respiration, skin conductance (and pulse oximeter signals) were collected simultaneously with a sampling rate of 5000 Hz using the 16-channel BrainAMP ExG MR (Brain Products GmbH, Gilching, Germany) amplifier and the BrainVision Recorder (Software version 1.22.0101, Brain Products GmbH, Gilching, Germany). Synchronization with the eye-tracker (non-constant sampling rate of about 250 Hz) and the presentation of pictures was done with common timestamps via the lab streaming layer (LSL) system in Lab Recorder (Swartz Center for Computational Neuroscience, UCSD; https://github.com/sccn/labstreaminglayer, accessed in July 2019). All settings of the recording software (BrainVision Recorder and Pupil Capture) were kept as default.

### Questionnaires

Before coming to the experimental site, participants completed three questionnaires to measure the severity of their fear of spiders: the FSQ, the SPQ and the four-item screening SAS, all in their German versions. Additionally, participants completed the German Trait-section of the State-Trait Anxiety Inventory. Immediately after the experiment, participants completed the FSQ and SAS questionnaires again. During the experiment, participants filled in four identical questionnaires (‘break questionnaires’) assessing their current degree of fear, disgust, physical excitement, boredom and exhaustion, each on a scale of 0 to 10, one after each of the four experimental blocks (or runs) (see Fig. 1).

## Data Records

The dataset can be found in the following OSF repository: https://osf.io/98cnd^23^. Additionally, the spider stimuli used to trigger the physiological responses are available at https://osf.io/vmuza^22^ in the ‘spiderPhy_luminance_corrected’ folder. The images in the ‘spiderPhy_luminance_corrected’ folder are licensed under CC BY-NC-SA.

Of note, the remaining folders of the https://osf.io/vmuza^22^ repository contain distinct material related to spider fear and are licensed under CC BY. The ‘spider_fMRI’ and ‘spider_all’ folders contain stimuli and fMRI material described in^24^. The ‘computerized_BAT’ folder contains the stimuli and experimental script of a computerized behavioral avoidance test described in^25^.

### Physiological signals

We provide one physiological data file per participant in.xdf format and in.mat format (converted from xdf), i.e., “ID_0xx_lsl_data.mat”.

The.mat file contains three Matlab structures, one for the experimental markers (called “fear_stream”), one for the eye-tracking data and one for BrainVision data (i.e., pulse, respiration, ECG and SCR). The experimental markers encode the onsets and offsets of each event as indices and their corresponding timestamps. The eye-tracking data contains 22 channels (one for confidence level, two (left and right) for raw pupil size, two (left and right) for 3D reconstructed pupil size and seventeen other channels for gaze and position). The BrainVision structures contains the signals acquired via the amplifier, with one channel for pulse, respiration, ECG and SCR. Signals are stored as “time_series”; timestamps, synchronized over all the signals, as “time_stamps”; other metadata such as sampling rates in “infos”.

A detailed description of the variables and of the structure of the Matlab files can be found in the OSF repository ‘readme.txt’.

### Stimuli ratings and trial onset data

The rating of each trial can be found in the.csv files (one file per participant, e.g., ‘ID0xx_ 2019_Jan_01_0101.csv’). The file lists (1) the images in the order by which they were presented (‘image n°), (2) the filename of the picture that was presented (‘picture ID’), (3) the fear rating given to that trial by the participant (‘rating’), (4) the reaction time to give the rating (or to perform the catch trial) (‘reaction time’) in seconds, and (5) the jittered duration of the baseline that precedes the presentation of the stimulus (‘cross jitter’) in seconds.

### Psychometric data

We provide demographical information (gender, age, education level, occupational category), personal information (motivation levels, vision) and questionnaire data (FSQ, STAI, SAS, SPQ sum scores) as well as self-reported psychological state data (fear, agitation, disgust, boredom, and exhaustion on a 0–10 scale) acquired during three short breaks in-between the exposure experiment. This data, as well as a description of each variable, are provided as an excel sheet ‘spiderPhy_beh_psy.xlsx’ in the OSF repository.

## Technical Validation

To substantiate the technical validity of the physiological signals, we provide a statistical comparison between responses to stimuli rated as high fear (first quartile) and the stimuli rated as low fear (last quartile).

### Preprocessing

**Fig. 2:**
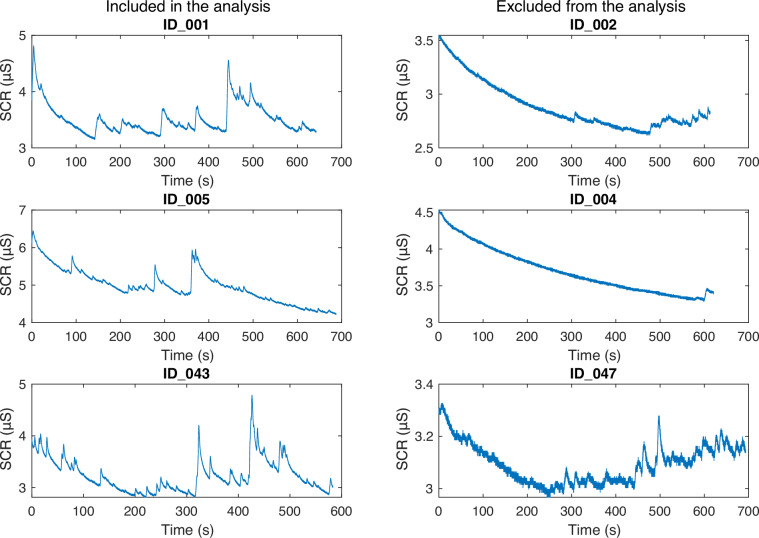
Illustrative examples of skin conductance responses (raw signals) that were considered of sufficient quality to be included in this analysis (left side) vs. those which were excluded (right side).

The signals first underwent visual inspection from 3 experimenters to screen out low-quality signals. After this step, we excluded 29 participants from skin conductance analysis (remaining N = 24) and none for respiration, pupil size and ECG (remaining N = 54). The reason for exclusion was the lack of visible tonic response in the SCR signals and/or high noise levels in the raw data. For practical reasons, our exclusion rule was done conservatively, with one low-quality run leading to the exclusion of the participant. As a result, we still included the signals of all the participants in this dataset to allow the flexibility of exclusion criteria in future analyses and to enable the recovery of the excluded signals. We provide an exclusion sheet in which we marked which participant was excluded in this analysis. Some examples of what we considered to be “good” vs. “bad” signals for the present analysis are provided in Fig. 2. Our main analyses were performed using the PsPM toolbox Version 6.0.0^26^ which ran on Matlab R2022a. All the signals were divided into 4 runs by trimming out the break periods starting from the first fixation cross of each run and ending with the end of the last rating window.

#### ECG

We first pre-processed the signals by applying a fourth-order Butterworth bandpass filter with a passband of 8–20 Hz (Matlab’s “designfilt” function), imported to PsPM, and converted ECG signals to Heart Period using the Pan & Tompkins method^27^ as implemented in the toolbox.

#### SCR

We applied a second-order Butterworth low-pass filter with a cut-off frequency of 30 Hz.

#### Pupil size

Since pupil size data was initially recorded at a variable sampling rate, we resampled the pupil dilation signal to a constant 250 Hz rate (Matlab’s “pchip” function). Since the eye-tracker software marks all sample points with a confidence value, we also applied the resampling step to this confidence signal as well and marked as NaN any point with low confidence (<0.6, i.e., the threshold suggested by the manufacturer: https://docs.pupil-labs.com/core/software/pupil-capture). To identify outliers, we applied a criterion of 3 Median Absolute Deviations (MAD) above the median within a 40-second moving window and replaced the datapoints as NaNs. Subsequent pupil size preprocessing was conducted using the “pupil size preprocessing” method of PsPM toolbox with its default parameters, followed by a manual visual inspection to ensure signal quality.

#### Respiratory signal

We applied a fourth-order Butterworth low-pass filter with a cut-off frequency of 2 Hz and converted to respiration amplitude with the PSPM toolbox.

### First-level analysis

For the four signals (heart period, SCR, pupil size and respiration amplitude), we applied PsPM’s GLM method for first-level analysis (i.e., within subject analysis) which mimics fMRI’s SPM standard method for data analysis. For each run, we specified one regressor for the first, second, third and fourth quartiles of the individual ratings of that run, plus one regressor for the catch trials. Each regressor was concatenated over runs and convolved with the built-in canonical response functions of the toolbox (with the first-order derivatives) respective to each signal, so that we obtain a beta estimate per fear level that derives from the trials of all 4 runs. Each signal was additionally filtered according to the default settings of the toolbox^26,28–30^.

### Second-level analysis

**Fig. 3:**
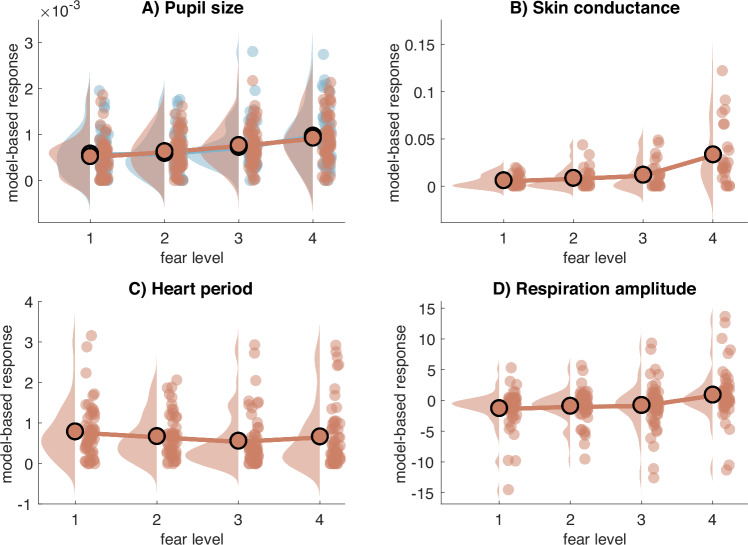
Model-based physiological responses to different fear levels. Each response was computed using a GLM-based approach as implemented in the PSPM toolbox. For each subject, we specified one regressor for each of the four levels, plus one regressor for catch trials. For pupil size (**A**), skin conductance (**B**) and heart period (**C**), each data point corresponds to the maximum of the response for each fear regressor, with one data point per subject. For respiration amplitude (**D**), since the response is biphasic, each data point corresponds to the peak of the early response. For all physiological measures, we found a significant difference between low-fear (first quartile) and high-fear trials (last quartile).

Finally, we extracted the GLM’s reconstructed responses (PsPM’s “pspm_glm_recon” function) of each fear quartile for each participant (N = 54 for ECG, pupil size and respiration amplitude; N = 24 for SCR). To interpret GLM-based signal responses, several metrics have been shown as relevant for capturing fear processing^26,28,29,31–34^. Here, for pupil size, heart period and skin conductance responses, we opted for maximum of the average response per subject^28,30,34^. For respiration amplitude, since the responses are biphasic^31^, we chose to compute the signed peak value between 1–7 s after stimulus onset to obtain the early average response. We used a two-tailed Wilcoxon Signed Rank Test to compare the model-based responses to low-fear stimuli (first quartile) and high-fear stimuli (last quartile). The Wilcoxon Signed Rank Test indicated statistically significant differences between high-fear and low-fear responses for all the signals: for pupil size *z* = 4.3246 (*p* < 0.0001), for skin conductance *z* = 3.8286 (*p* = 0.0001), for respiration amplitude *z* = 4.163 (*p* < 0.0001), and for heart period *z* = −2.0277 (*p* = 0.04) (Fig. 3). These results are in line with previous research on physiological responses to fear or emotional stimuli^35–37^.

## Data Availability

PsychoPy presentation scripts and analysis scripts are available at https://osf.io/98cnd/^23^.
